# An insight on sources and biodegradation of bioplastics: a review

**DOI:** 10.1007/s13205-023-03638-4

**Published:** 2023-05-31

**Authors:** Nag Pooja, Ishita Chakraborty, Md. Hafizur Rahman, Nirmal Mazumder

**Affiliations:** 1 Department of Biophysics, Manipal School of Life Sciences, Manipal Academy of Higher Education, Manipal, Karnataka 576104 India; 2 Department of Quality Control and Safety Management, Faculty of Food Sciences and Safety, Khulna Agricultural University, Khulna, Bangladesh

**Keywords:** Bioplastics, Biodegradation, Starch, Cellulose, Conventional plastics

## Abstract

Durability and affordability are two main reasons for the widespread consumption of plastic in the world. However, the inability of these materials to undergo degradation has become a significant threat to the environment and human health To address this issue, bioplastics have emerged as a promising alternative. Bioplastics are obtained from renewable and sustainable biomass and have a lower carbon footprint and emit fewer greenhouse gases than petroleum-based plastics. The use of these bioplastics sourced from renewable biomass can also reduce the dependency on fossil fuels, which are limited in availability. This review provides an elaborate comparison of biodegradation rates of potential bioplastics in soil from various sources such as biomass, microorganisms, and monomers. These bioplastics show great potential as a replacement for conventional plastics due to their biodegradable and diverse properties.

## Introduction

Plastics are crucial materials in modern life, and due to their resistance to chemical, physical and biological degradation, society relies majorly on them (Bhogayata and Arora 2018). However, the overexploitation of plastics by the ever-growing human society has led to various environmental and health risks (Wright and Kelly 2017; Bradney et al. 2019). Two of the main factors why plastics are so widely used are their durability and inexpensiveness, allowing them to be used for various applications like food preservation, packaging, transportation, building, construction, etc. Unfortunately, the poor post-production management of plastics becomes a significant problem in aggravating its impact on the environment. The inefficient waste management and deliberate littering have resulted in tons of plastic waste floating in the oceans, causing damage to marine ecosystems. Global petroleum-based plastic production rose from 1.7 million tons in 1950 to 322 million tons in 2015 (Suman et al. 2020). It was also estimated that due to the increased use of plastic personal protective gear during the COVID-19 pandemic, 585 million tons of single-use plastic waste would be generated by the end of 2020. The major contributing countries to generating plastic waste are China, India, the USA, and Brazil (Benson et al. 2021). This demands an alternative to the ubiquitous plastics, and bioplastics have emerged as promising alternatives. ‘Biodegradable bioplastics’ are plastics that are derived from renewable biomass which are bio-based such as starch, cellulose, collagen, polylactic acid, and polyesteramides (Coppola et al. 2021). The use of bioplastics can reduce the dependency on fossil fuels which are present in limited amounts. Unlike petroleum-based plastics, bioplastics emit a lesser amount of greenhouse gases (Mittal et al. 2022). The term bioplastic does not necessarily mean that they are biodegradable or eco-friendlier. Some of the bioplastics are not biodegradable or may require a very long time to disintegrate. Bio-polyethylene (bio-PE), bio-polypropylene (bio-PP), bio-polyethylene-terephthalate (bio-PET), bio-polytrimethylene terephthalate (Bio-PTT), and bio-polyamide (bio-PA) are some of the most common non-biodegradable bioplastics (Rahman and Bhoi 2021). Biodegradable polymers are defined as polymers that can be degraded into carbon dioxide, water, methane, and other low-molecular-weight compounds (Ishigaki et al. 2004).

In biodegradation assays, two fundamental approaches are used: aerobic and anaerobic digestion (Ruggero et al. 2019). Biodegradation is a set of chemical reactions that take place in the presence of living organisms such as bacteria, fungi, yeast, algae, and insects under optimum light, temperature, and oxygen. The degradation process is also influenced by the microstructure of the polymer. Furthermore, the rate of biodegradation might differ, depending on the environmental conditions and the polymer’s integral molecular structure (Scaffaro et al. 2019). Most of the plastic wastes are dumped as landfills and in soil, leading to the emission of greenhouse gases and leachate. Using compost in the degradation of different biodegradable bioplastics has been studied. Composting is classified into three types: aerobic, anaerobic, and vermicomposting. Aerobic composting occurs in the presence of plenty of oxygen. Aerobic microorganisms degrade organic matter, producing carbon dioxide (CO_2_), ammonia, water, heat, and humus. Anaerobic composting involves decomposition in the absence or limited supply of oxygen. Anaerobic microorganisms produce intermediate compounds such as methane, organic acids, and hydrogen sulfide. Vermicomposting is the decomposition process that involves the use of various species of earthworms to produce a decomposition mixture (Jouhara et al. 2017; Sanchez-Hernandez et al. 2020). However, composting rates at home and in industrial conditions may differ. Home composting is a basic process that creates nutrient-rich soil by decomposing organic waste such as vegetable waste and food leftovers in compost bins. However, the conditions and temperatures for home composting are not efficient in breaking down bioplastics like PLA. Industrial composting on the other hand provides a strictly monitored composting process that includes measured inputs of water, air, and carbon and nitrogen-rich materials to ensure rapid biodegradation of organic material. The residues produced can be integrated into the natural geochemical cycle (Schrader et al. 2017; Narancic et al. 2018). Further, most industrial composting sites have leachate collection and storage systems where the stored leachate can be pumped into the wastewater treatment plant for further processing. This will prevent the leachate from running into the freshwater bodies and causing eutrophication.

Biodegradability is the foremost important factor to be considered when bioplastics are considered over regular plastics (Din et al. 2020). Bioplastics from various sources exhibit different mechanisms and rates of biodegradation. Biodegradation also depends on several aspects such as environmental factors, moisture, and microbial presence. Extracellular enzymes secreted by microorganisms assist the biodegradation of polymers in processes involving the hydrolysis of ester linkages to release monomers. Both biotic and abiotic processes influence the total breakdown of organic matter. Microorganisms break down the material through biotic decomposition, while abiotic processes such as photodegradation and chemical hydrolysis break down the material chemically and physically at high temperatures and/or under acidic or basic pH conditions. (Polman et al. 2021). There is a lack of reviews that study and compare the detailed biodegradation of bioplastics from all the common sources. This review aims to provide a comprehensive insight into the commonly used sources of bioplastics and several factors affecting their biodegradation. Sources of Bioplastics” elaborates on the broad classification of bioplastics based on the source of raw materials. Some of the most widely used sources for the synthesis of bioplastics like polysaccharides (starch, cellulose, chitin and pectin), proteins (collagen, whey), polyhydroxyalkanoates and other sources such as polylactic acid, polyesteramides have been reviewed. Table 1 summarizes the sources, advantages, and disadvantages of various native biodegradable bioplastics. “Biodegradation of bioplastics” includes the process of biodegradation of the most commonly used bioplastics. The microorganisms and enzymes involved in their degradation are enlisted in Table 2. A summary of the percentage of degradation of bioplastics from different sources is provided in Table 3.

**Table 1:** Summary of sources, advantages, and disadvantages of various native biodegradable bioplastics

Raw material	Origin	Advantages	Disadvantages	References
Polysaccharides				
Starch	Corn, potato, rice, tapioca, tapioca/cassava, banana, wheat, yam, sago and buckwheat	• Good film forming properties, oxygen and aroma permeability	• Brittleness • Poor mechanical strength • Hydrophilicity	(Thakur et al. 2019)
Cellulose	Cotton, wood, sisal, flax, hemp, jute, sugarcane bagasse	• Ease of availability • Low cost of production • Transparency	• Susceptible to moisture	(Silva et al. 2016)
Lignocellulose	Wood pulp, jute, hemp, cotton	• Antimicrobial properties • Good viscoelastic, film-forming capacity	• Hydrophilicity • Incompatibility with many polymers • Brittleness	(Wang et al. 2011)
Pectin	Apples, guavas, citrus fruits, plums	• Ease of availability	• Poor mechanical properties • Hydrophilicity	(Liu et al. 2005)
Chitosan	Chitin (exoskeleton of crustaceans like crabs, lobsters, prawns, shrimps)	• Inherent antimicrobial and antifungal activity • Good mechanical strength • Low oxygen and carbon dioxide permeability	• High water sensitivity	(Martínez-Camacho et al. 2013)
Proteins				
Casein	Milk, cheese, yogurt, and other dairy products	• High thermal stability	• Difficulty in moldability	(Dodd 2010)
Whey protein isolate	Waste stream of the cheese industry	• Good oxygen and aroma permeability	• Moderate moisture • barrier capacity • Require plasticizers to create easy to handle films	(Galus and Kadzińska 2016)
Collagen	Fish, chicken, egg whites, seafood	• Good oxygen and aroma barrier capacities	• Relatively low water barrier capacity • Poor mechanical strength	(Ma et al. 2018)
Zein	Corn	• Good film-forming properties after dissolving in ethanol and acetone • Good tensile strength and moisture barrier properties	• Brittleness	(Ghanbarzadeh et al. 2006)
Soy protein isolate	Soybean	• Transparent and flexible films • Gas barrier properties	• Poor mechanical properties • High sensitivity to water	(Zheng et al. 2017)
Gluten	Waste stream of the wheat starch industry	• Low cost • Good oxygen barrier capacity • Good film-forming properties	• High moisture sensitivity • Brittleness	(Zhang et al. 1996)
Microorganisms				
PHAs	*Ralstonia eutropha*	• Excellent barrier capacity to carbon dioxide, oxygen, and water • Good water resistance	• Difficulty in sustaining optimal growth conditions • High cost of recovery	(Zakaria Gomaa 2014)
Biobased monomers				
PLA	Lactic acid	• Excellent film-forming properties • Hydrophobic	• Brittleness and rigidity • Incompatibility with certain polymers	(Elsawy et al. 2017)
Synthetic monomer				
PCL	ε-caprolactone	• Hydrophobic • Oil, solvent, and chlorine resistance	• Low melting point	(Dong and Walker 2012)
PBAT	Adipic acid, 1,4-butanediol, and terephthalic acid	• Flexible • Good tensile strength	• Low thermal stability • Stiffness	(Al-Itry et al. 2012)
PEA	Hydrophobic α-amino acids, α, ω-diols, aliphatic dicarboxylic acids and dianhydrohexitoles	• High thermal stability and tensile strength	• Expensive	(Zou et al. 2004)

*PLA* Polylactic acid; *PHA* Polyhydroxyalkanoates; *PCL* Polycaprolactone; *PBAT* Polybutylene adipate terephthalate; *PEA* Polyesteramides

**Table 2:** Summary of microbes and enzymes responsible for degradation of biodegradable biopolymers

Biodegradable polymer source	Microorganisms		Enzymes	Reference
	Bacteria	Fungi		
Polysaccharides				
Starch	*Bacillu*s sp: *B. amyloliquefaciens*, *B. subtilis,* *B. licheniformis*, *B.* *stearothermophilus,* *B. megaterium*, *B. circulans*	*Clonostachys rosea, Trichoderma sp*	Amylase • α amylase; endoamylase (EC 3.2.1.1) • β amylase; exoamylase (EC 3.2.1.2) • Glucoamylase (EC 3.2.1.3)	(Alariya et al. 2013; Urbanek et al. 2017; Luang-in et al. 2019)
Cellulose	*Clostridium*, *Cellulomonas*, *Cellulosimicrobium*, *Thermomonospora*, *Bacillus*, *Ruminococcus*, *Erwinia*, *Bacteriodes*, *Acetovibrio*, *Streptomyces*, *Microbispora*, *Fibrobacter*, and *Paenibacillus*	*Trichoderma*, *Aspergillus*, *Penicillium*, *Phanerochaete,* and* Fomitopsis*	Cellulases: • β-1,4-endoglucanase (EC 3.2.1.4) • Cellobiohydrolase or exoglucanase (EC. 3.2.1.91) • β-glucosidase or cellobiase (EC. 3.2.1.21	(Liang et al. 2014; Fernandes et al. 2018)
Lignocellulose	*Streptomycesviridosporus T7A, Nocardia autotrophica, Sphingobium sp. SYK-6,* *Pseudomonas putida mt2, Rhodococcus sp.,* *Burkholderia cepacia, Microbacterium sp., and Citrobacter sp*	*Basidiomycetes, Ascomycetes*	Ligninolytic enzymes: • Laccase (EC 1.10.3.2) • Lignin peroxidase (EC 1.11.1.14) • Manganese peroxidase (EC 1.11.1.13)	(Bugg et al. 2011; Kumar and Chandra 2020)
Chitin	*Streptomyces*, *Alteromonas Escherichia, Aeromonas, Serratia marcescens*	*A. nidulans, N. crassa, M. oryzae, Schizosaccharomyces pombe, S. cerevisiae, A. oligospora*	Chitinases: Glycosyl hydrolases (E.C 3.2.2.14)	(Yang and Zhang; Beier and Bertilsson 2013; Hamid et al. 2013)
Pectin	*Streptomyces sp, Cellulomonas sp, Bacillus sp*	*Aspergillus fumigatus, A. flavus, A. niger, A. ochraceus, A. oryzae, A. sydowii, Trichothecium sp., Penicillium sp., Trichoderma harzianum and T. viride*	Pectinases (EC 3.2.1.15): • Pectin methyl esterase (EC 3.1.1.11) • Polygalacturonases (endo-, EC 3.2.1.15; exo-, EC 3.2.1.67) • Pectin transeliminases (EC 4.2.2.10)	(Ramos 2011; Shrestha et al. 2021)
Proteins				
Casein, whey, soy protein, gluten	*Bacillus sp*	*Penicillium, Aspergillus, Rhizopus, Endothia, Mucor*	Proteases /Proteolytic enzymes	(Razzaq et al. 2019)
Microorganisms				
PHAs	*Enterobacter sp, Bacillus sp, Gracilibacillus sp.,* *Pseudomonas lemoigne, Comamonas sp.* *Acidovorax faecalis, Aspergillus fumigatus,* *Variovorax paradoxus,* *Streptomyces sp,* *Aspergillus sp.*	*Penicillium pinophilium, Penicillium funiculosum, Paecilomyces lilacinus, Aspergillus fumigatus, Emericellopsis minima*	Polyhydroxyalkanoate (PHA) depolymerase (EC 3.1.1.75)	(Volova et al. 2010; Singh et al. 2020) (Tokiwa et al. 2009)
Bioderived monomers				
PLA	*Bacillus pumilus*	*Tritirachium album,* *Fusarium moniliforme, Penicillium roqueforti, Clonostachys rosea and Trichoderma sp*	• Carboxylesterase (EC 3.1.1.1) • Cutinases (EC 3.1.1.74) • Lipases (E.C. 3.1.1.3) • Serine proteases (EC 3.4.21.14)	(Tokiwa et al. 2009; Bonifer et al. 2019; Butbunchu and Pathom-aree 2019)
PBAT	*Thermobifida fusca, Pelosinus fermentan,s* *lostridium botulinum, Pseudomonas sp*		• Carboxylesterase (EC 3.1.1.1) • Lipases (E.C. 3.1.1.3)	(Wallace et al. 2017) (Tokiwa et al. 2009; Singh et al. 2020)
PEA	*Aspergillus, Aureobasidium, Penicillium, Pullularia*	*Penicillium* sp, *R. arrizus, R.delemar, Achromobacter* sp. and *Candida cylindracea*		
PCL	*Clostridium sp*	*Aureobasidium sp., Cryptococcus sp., Aspergillus flavus, A. niger, A. fumigatus, Chaetomium globosum, Pencillium funiculosum, Fusarium sp.* *Clonostachys rosea, Trichoderma sp*.		

*PHA* Polyhydroxyalkanoates, *PLA* Polylactic acid, *PBAT* Polybutylene adipate terephthalate, *PEA* Polyesteramides, PCL Polycaprolactone

**Table 3:** Table comparing the percentage of biodegradation of some bioplastics from various source

Sources	Mass loss (%)	Conditions of biodegradation	Approx. Duration (days)	Reference
Biomass				
Polysaccharides				
Starch	~ 90	Organic compost Moisture content: 50% pH: 7.0–8.0	31	(Torres et al. 2011)
Cellulose	~ 30		31	
Chitin	83.8	Burial in red clay	31	(NAKASHIMA et al. 2005)
Lignin	19–60	Laboratory incubation	91- 730	(Zabel and Morrell 2020)
Pectin-cellulose composite	90	Anaerobic digestion in batch reactors maintained at 55 °C	15	(Bátori et al. 2017)
Protein				
Soy protein	30	Aqueous solution containing pronase (proteolytic enzyme)	6	(Yamada et al. 2020)
Casein	20	Loam soil	7	(Bagares et al. 2020)
Zein	58	Saturated field soil with Fafard® 52 medium	84	(Helgeson et al. 2009)
Soy protein and whey protein isolate composite	36	Composting	7	(Li and Chen 2000)
Microorganisms				
PHA	88–99	Under optimum static laboratory conditions	49	(Siracusa et al. 2008)
PHB	62	Fertile garden soil with pH 7.30 and humidity of 80% at 30 °C	62	(Altaee et al. 2016)
PHB–TiO_2_ composite nanofiber films	100		21	
PHBV	100	Lab-scale composting	60–80	(Salomez et al. 2019)
Monomers				
Bio-derived monomers				
PLA	88	Composting	90	(Kawashima et al. 2021)
Synthetic monomers				
PCL	92	pH 13	63	(Sailema-Palate et al. 2016)
	80	pH 1	69	
	100	Composting	1460	(Manoukian et al. 2019)
PBAT	2.38	Real soil burial	91	(Wang et al. 2015)

*PHA* Polyhydroxyalkanoate, *PHB* Polyhydroxybutyrate, *TiO*_*2*_ Titanium dioxide, *PHBV* Poly(3-hydroxybutyrate-co-3-hydroxy valerate, *PLA* Polylactic acid, *PCL* Polycaprolactone, *PBAT* Polybutylene adipate terephthalate

## Sources of bioplastics

**Fig. 1:**
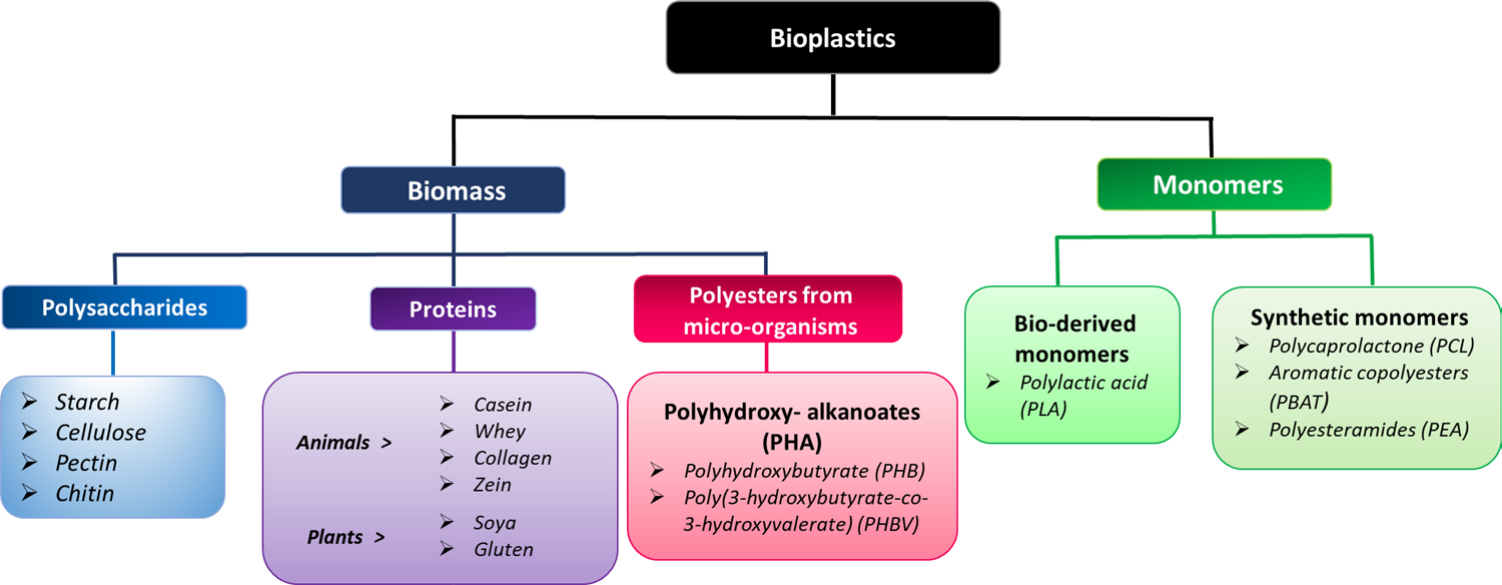
Classification of bioplastics based on sources (biomass, microorganisms, and monomers). The figure is adapted from (Avérous 2004) with kind permission from Taylor & Francis

Broadly, bioplastics are produced from biopolymers obtained from biomass, and monomers. Bioplastics are classified based on their source of raw material as depicted in Fig. 1 and are discussed below.

### Biomass

Bioplastics made from renewable biomass have sparked curiosity in recent years. Polysaccharides including starch, cellulose, chitin, pectin as well as proteins are extracted from conventional animal and plant sources.

#### Polysaccharides

Polysaccharides, including starch and cellulose, are the primary and most prevalent biobased polymers utilized to manufacture bioplastics (Lubis and Harahap 2018; Abe and Branciforti 2021). Other lesser-known polysaccharides such as chitin and pectin are also used. (Tables 1 and 3). Starch is a highly degradable, most abundant homopolysaccharide present in plants, consisting of both linear (amylose) and branched (amylopectin) structures. Corn, potatoes, banana, tapioca, wheat, rice, yam, sago, and buckwheat are the traditional sources of starch used in the production of bioplastics (Marichelvam et al. 2019; Abral et al. 2019; Jiménez-Rosado et al. 2019; Asrofi et al. 2020). The synthesis of starch-based bioplastics involves heating native or modified starch in the presence of water to initiate gelatinization. As much as fifty percent of commercially available bioplastics are produced from starch (Erabela et al. 2021). Due to their simple and cost-effective production process, starch-based bioplastics have a broad range of applications in the packaging industry (Liang and Wang 2018; Meereboer et al. 2020). Cellulose, another highly abundant homopolysaccharide consisting of repeating units of D-glucose bound together by β (1 → 4) glycosidic bonds is typically added to starch to enhance its mechanical properties, gas permeability, and water resistance. The most popular raw material used to produce cellulose-based bioplastics is softwood (Liu et al. 2019b). Cellulose is also used in different forms as a reinforcement filler in other biodegradable polymer matrices to improve their mechanical and physical properties. Lignin is a complex plant-based polymer found in vascular tissues as a supporting structure. It is called lignocellulose since it is found in cell walls alongside cellulose and hemicellulose. The composition of lignin in the lignocellulose varies according to the botanical source (Le Digabel and Avérous 2006). Lately, lignocellulosic fibers are being utilized as bioplastic reinforcements in place of synthetic fibers due to their ability to be biodegraded and renewable. A significant number of studies have focused on combining lignin with other bio-based polymers like starch, cellulose, and polylactic acid (PLA) to synthesize biodegradable bioplastics (Kai et al. 2016; Shi and Li 2016; Brodin et al. 2017). Pectin is a heteropolysaccharide distributed in the primary lamella, middle lamella, and cell walls of plants. It is made up of esterified d-galacturonic acid units bound together by α-(1–4) glycosidic bonds (Ropartz and Ralet 2020). Citrus fruits like oranges, lemons, gooseberries, and strawberries are rich in pectin, which was used to develop biodegradable bioplastics for the food and packaging industries. However, its applications are limited due to its inadequate mechanical properties and hydrophilic nature. (Shrestha et al. 2021). Like other polymers, several studies reported the addition of plasticizers like polyols to help in tackling the brittleness of the films. Also, pectin is blended with different additives like sodium alginate, starch, cellulose, and chitosan to improve the properties of the resulting bioplastic (Nešić et al. 2017; Younis and Zhao 2019). Chitin makes up the exoskeleton of crustaceans and is one of the most abundant polysaccharides. Chitin undergoes N-deacetylation in an alkaline medium to form chitosan, a linear molecule composed of β- (1 → 4) -linked d-glucosamine and N-acetyl-d-glucosamine. Owing to its non-toxicity and biodegradability, chitosan was extensively studied for its ability to form environment-friendly plastics (Table 1). It is one of the few polysaccharides known for its native antimicrobial properties against gram-negative, gram-positive bacteria, algae, and fungi. Temperature is also a crucial factor in evaluating the antimicrobial activity of chitosan-based films/bioplastics, according to a report (Leceta et al. 2013). The film-forming solution showed antibacterial activity but was only found to be bacteriostatic after being dried into films. The use of various polyols, like glycerol, sorbitol, and polyethylene glycol as plasticizers for chitosan is studied extensively. The physicochemical properties of the film are influenced by the type and volume of plasticizer used (Ma et al. 2019; Jha 2020).

#### Proteins

Many proteins, of both plant and animal origin, are used as viable options for the creation of packaging materials.

##### Proteins of animal origin

Casein is a milk protein belonging to the family of phosphoproteins and is majorly found in mammalian milk (Shivani et al. 2021). To form a bioplastic, casein is dispersed in an aqueous alkali solution, followed by coagulation with an acid or salt of an acid (Yong et al. 2021). The resulting coagulum is pressed into plates and soaked in formaldehyde until it hardens to obtain the casein-based bioplastic. Rennet casein was extruded with water in the presence of high moisture and pressure to obtain pliable films. Despite its low cost, the film was not easily moldable, and as a result, it was phased out of commercial use (Dodd 2010). Furthermore, the formaldehyde used was a probable carcinogen that can cause genetic abnormalities, making its use hazardous (Jefferson et al. 2020). Whey protein is the by-product of cheese manufacturing, i.e., the liquid portion of milk after it is curdled and strained. The ability of whey to produce biodegradable, odorless, and transparent films are being extensively researched (Oliveira et al. 2017; Schmid and Müller 2018). The films are formed by casting and drying the whey protein isolate. But these films are highly brittle due to their disulfide crosslinking and are hence hydrophobic. To reduce the intermolecular interactions and overcome brittleness, it is treated with various plasticizers like glycerol and sorbitol. With the addition of plasticizers, the percentage of water solubility improved gradually, while mechanical resistance, Young’s modulus, and glass transition temperature decreased (Galietta et al. 1998). Collagen is another protein widely studied for its film-forming properties (Table 1). It is a structural protein present in the extracellular matrix of connective tissues like skin, cartilage, bones, and tendons. Collagen undergoes partial hydrolysis to form gelatin. It is commonly used in the fabrication of scaffolds, wound dressings, drug delivery systems, and packaging materials (Ge et al. 2018; Irawan et al. 2018; Lin et al. 2019; Bhuimbar et al. 2019). Collagen-based films are known for their excellent biocompatibility, biodegradability, and oxygen and aroma barrier capacities. However, the applications of these collagen-based films are limited in packaging due to their relatively low water barrier capacity and mechanical strength. To improve these properties, collagen is often combined with chitosan, soy protein, and reinforcement fillers such as zinc oxide nanocrystals, cellulose, and magnesium oxide nanoparticles (Ahmad et al. 2016; De Silva et al. 2017; Andonegi et al. 2020; Jiang et al. 2020).

##### Proteins of plant origin

Zein belongs to a group of plant storage proteins called prolamins, found majorly in the endosperm of maize (corn), accounting for 47% of the total endosperm content (dry basis) in corn (Shukla and Cheryan 2001). It is used in textile industries to impart hydrophobicity to the fabric. The hydrophobicity of the molecule is attributed to the recurrence of many non-polar amino acids like alanine, leucine, and proline in the structure. The fabric can also be rendered antimicrobial by encapsulating ellagic acid in zein molecules (Gonçalves et al. 2020). Zein also finds its applications in the production of ceramics for bone tissue repair (Hum et al. 2018), drug-delivery systems (Labib 2018), cosmetics (Tinoco et al. 2021), adhesives (Wei et al. 2020), etc. The plasticizing property of zein is widely studied and used in preparing thermostable, biodegradable zein-based bioplastics. The resulting zein-based bioplastic exhibits excellent gas barrier properties and hydrophobicity and is, therefore, used in modified atmosphere packaging (MAP) and coating on fruits and vegetables to preserve their freshness. MAP is a technique that involves the utilization of packaging films to control and modify the atmosphere around the packed product. The food is either sprayed, brushed, or dipped in the plasticized zein solution and allowed to solidify to form a thin coat (Neo et al. 2013). These bioplastic coatings improve the shelf life of the food products like apples, pear, mango, tomato, broccoli, rice, cheese, and roasted peanuts by altering certain processes like delaying respiratory rate, sprouting, germination, ripening, rancidity, reducing loss of moisture, and inhibiting microbial growth (Koh et al. 2018; Santos et al. 2018b, a; Zhang et al. 2019). Soy protein is a protein that is extracted from dehulled and defatted soybeans. The purified form of soy protein called the soy protein isolate (SPI) is obtained by alkaline suspension, followed by isoelectric precipitation at pH 4.5 (Chove et al. 2001). Attributed to the prevalence of polar functional groups such as hydroxyl, thiol, carboxyl, and amine in the molecule, the films are hydrophilic and have poor mechanical strength (Ye et al. 2019). This limitation can be overcome by performing certain surface modifications such as crosslinking with chemical agents like formaldehyde, glutaraldehyde, and phenolic compounds (Insaward et al. 2015). The resulting films from soy protein are brittle, hence various plasticizers are used to reduce this and improve the processability and flowability of the films (Božič et al. 2015). Improvement of SPI-based bioplastic properties is reported to be dependent on characteristics such as the size and polarity of the plasticizer used. Microbial contamination is another downside of the material. Several studies have used different nanoparticles and metal ions to impart antimicrobial activity to SPI-based films (Jin et al. 2020). A self-healing, antimicrobial, SPI bioplastic was developed with good mechanical strength using polyethyleneimine (PEI) and metal ions such as Cu^2+^ or Zn^2+^. It is believed that the polycationic property of PEI is responsible for disrupting bacterial membranes through ion exchange (Li et al. 2019). Several other studies have used different additives like cellulose nanocrystals, zinc oxide nanoparticles (Xiao et al. 2020), cortex philodendron extract (Liang and Wang 2018), grape seed extract, nisin, EDTA (Sivarooban et al. 2008), and organic acids like citric, lactic, maleic acids (Eswaranandam et al. 2004), etc. Gluten belongs to a family of seed proteins found in cereal grains like barley, rye, and majorly in wheat. The total wet and dry gluten content of wheat ranges from 17.8 to 47.23% and 5.9 to 10.1% respectively. The viscoelastic property of gluten is exploited in the synthesis of biodegradable plastics. Factors like the production technique, working duration, temperature, pH, plasticizer, and additive content can influence the properties of the film (Yu et al. 2016). It was reported that gluten-based films obtained through extrusion were found to have better plasticizer–gluten interaction and water uptake capacity when compared to that produced through compression molding. The study also reported that an increase in pH toward the alkaline side improved the water uptake capacity of the wheat gluten films whereas those with additives like xanthan gum and glyoxal showed lower water uptake (Jiménez-Rosado et al. 2019). Proteins are heteropolymers, unlike many other biodegradable polymers discussed above. The presence of various amino acids provides a wide range of chemical functions, which can result in a broad spectrum of polymer network architectures. Proteins such as gluten provide unique and favorable properties like viscoelasticity and flow properties to the developed bioplastic (Table 1).

#### Polyesters of bacterial origin

Polyesters are polymers made up of a carboxylic acid and diol group (Sudesh et al. 2000). A variety of bacteria that have been grown under various nutrient and environmental conditions, produce polyesters. These molecules, which are mainly lipid-based, are collected as storage resources, facilitating microbial survival in stressful situations. Based on the source organism, the granular size and number, macromolecular structure, monomer composition, and physicochemical characteristics change (Luengo et al. 2003). Most of these substances have the ability to break down naturally and are compatible with living organisms, making them highly interesting to the biotechnology industry. When provided with vital nutrients such as oxygen, phosphorus, nitrogen, sulfur, and magnesium, bacteria can proliferate rapidly. However, during the prevalence of imbalance in the growth environment, i.e., in the excess of carbon source and deficiency of essential nutrients, mainly phosphorus, and nitrogen, a variety of bacterial and archaeal genera accumulate storage polymers named polyhydroxyalkanoates (PHAs) (Wong et al. 2012). One of the most thoroughly researched microorganisms for the manufacture of PHA is *Ralstonia eutropha* or *Alcaligenes eutrophus.* They are synthesized and accumulated as highly refractive granules in the cytoplasm of the microorganisms. PHA acts as a carbon and energy reserve helping in the survival of bacteria under nutrient-deficient conditions (Sirohi et al. 2021). The bacteria also show enhanced stress tolerance toward ultraviolet (UV) irradiation, heat, and osmotic shock (Kadouri et al. 2005). Pertaining to the microbe and growth conditions, different types of PHAs are synthesized. More than 150 types of PHAs have been identified so far. Polyhydroxybutyrate (PHB) is a well-known polymer belonging to the PHA family. Other members include polyhydroxy valerate (PHV) and poly (3-hydroxybutyrate-co-3-hydroxy valerate) (PHBV). PHA is recovered from the bacterial cell through lysis of the cell wall, followed by solubilization, purification, and precipitation of PHA polymer (Kunasundari and Sudesh 2011). PHAs are biodegradable, non-toxic, biocompatible, water-resistant, and highly crystalline. Owing to their novel features, PHA bioplastics are used in the manufacture of biodegradable packaging materials (Meereboer et al. 2020). The need to maintain optimal growth conditions and the high cost of recovery are the limitations of PHA bioplastics. However, the utilization of recombinant microorganisms can overcome these disadvantages (Carpine et al. 2020).

### Monomers

Several monomers (both naturally occurring (bio-derived) and synthetic) are combined by the process of polymerization to produce efficient bioplastics. Overall, the choice of monomer depends on factors such as the properties required for the final product, the cost of production, and the availability of renewable resources.

#### Bio-derived monomers

Polylactic acid (PLA) is an aliphatic polyester formed by condensation of lactic acid. It can also be obtained by the ring-opening polymerization of lactide (Mehta et al. 2005). PLA has been hailed as a promising, environment-friendly polymer due to its low toxicity and sustainability (Mulvihill et al. 2011). Like any other polymers, PLA films have a high modulus and are brittle (Xu et al. 2020). The use of plasticizers reduces the intermolecular forces of the polymeric chains, resulting in better processability, flexibility, and ductility. Citrate, adipate, and oligomeric lactic acid are by far the most widely used plasticizers for PLA (Shirai et al. 2015; Singh et al. 2019). Plasticizers from packaging materials tend to migrate into food over time, posing a risk to one's health. To delay the migration of plasticizers, fillers like wheat bran and nano-additives such as chitin nanofibrils (CN) are added to the blend. A study was carried out to investigate the ability of chitin nanofibrils and calcium carbonate (both micrometric and nanometric) in preventing or controlling the leaching of the plasticizer acetyl n-tributyl citrate (ATBC) in PLA/PBS blends. It was found that the addition of both chitin nanofibrils and micrometric calcium carbonate helped to slow down the leaching of the plasticizer (Aliotta et al. 2020, 2022). Melt mixing PLA with other polymers such as polycaprolactone (PCL), polybutylene succinate (PBS), starch, polyhydroxy butyrate (PHB), polyvinyl alcohol (PVOH), and polybutylene adipate terephthalate (PBAT) is another way to boost its mechanical properties (Anna and Arrigo 2019; Olaiya et al. 2019; Kahvand and Fasihi 2019; Xiang et al. 2020). In conclusion, one of the most appealing features of PLA plastic is that its properties can be modified by additives, expanding its potential applications (Table 1). Additionally, PLA is biodegradable under environmental conditions. However, PLA polymers do have some limitations, such as softening at 60 °C. Copolymerization with a more heat-resistant polymer or the incorporation of fillers can aid in enhancing its thermal stability. 

#### 2.2.2. Synthetic monomers

Polycaprolactone (PCL) is a biodegradable polyester derived from fossil or crude oil. It was one of the first synthetic polymers to be developed. It is obtained from the monomer ε-caprolactone employing ring-opening polymerization. PCL is known for its hydrophobicity, flexibility, mechanical strength, excellent blend compatibility, and biodegradability (Woodruff and Hutmacher 2010). It is used in the synthesis of polyurethanes and as biodegradable, hot melt adhesives (HMAs) in food packaging. It is also gaining popularity in the biomedical field in the fabrication of biodegradable scaffolds, drug delivery systems, wound dressings, and contraceptive devices (Kim and Kim 2014; Siddiqui et al. 2018). Among the aromatic co-polyesters, polybutylene adipate terephthalate (PBAT) is one of the most promising materials with great potential in various applications (Table 1). It is a biodegradable, synthetic, block copolymer of adipic acid, 1,4-butanediol, and terephthalic acid (Jian et al. 2020). It is manufactured under different commercial names such as Ecoflex, Origo-Bi, Ecoword, and Wango (Siegenthaler et al. 2012; Jacquel et al. 2015). PBAT has a random structure, hence the name random copolymer. The lack of structural order makes the material very stiff and of low elastic modulus; however, it is tough and flexible. The mechanical strength of the film depends on the composition of monomeric units and their molecular weight. Young's modulus increases with increasing terephthalate units while decreasing the elongation at break. Similarly, the tensile strength is positively correlated to the molecular weight while the elongation at break shows a negative correlation (Jian et al. 2020). The blend of PBAT and other strong, biodegradable polymers can yield robust and rigid films. Over the years, PBAT has found its use in cling films for packaging, mulch films, water-resistant coatings (Souza et al. 2020), etc. Polyester amides (PEA) are a new class of biodegradable polymers that are used in diverse industrial applications (Table 1). PEA can be produced from different monomers using a variety of synthesis techniques like ring-opening and polycondensation methods (melt, solution, and interfacial polycondensation (Rodriguez-Galan et al. 2011). These monomers have ester and amide linkages in their chemical structure that can be easily degraded by bacteria. Hydrophobic α-amino acids, α, $\omega$-diols, aliphatic dicarboxylic acids, and dianhydrohexitoles are some of the monomers used in the synthesis of PEA films (Gomurashvili et al. 2000). PEA combines the benefits of polyesters and polyamides in a polymer. The synthesized polymeric films possess good thermal, chemical resistance, and mechanical properties. Over the years, various fillers have been added to enhance the performance and durability of PEA films. A study reported that melt-blending of octadecyl amine-treated montmorillonite clay with PEA showed a slight improvement in the oxygen and water vapor barrier properties (Krook et al. 2002). Another study reported that the addition of nano-silicon dioxide and nano-calcium carbonate as fillers for PEA showed a significant increase in tensile strength and reduction in the rate of hydrolysis (Liu et al. 2007).

Biodegradable bioplastics from various sources mentioned above may have a wide variety of uses based on their respective properties such as texture, mechanical strength, hydrophilicity, gas barrier properties, biodegradability, and many more. Based on these properties, the developed bioplastics can be used for various applications from food packaging to textiles and biomedical applications. Thermoplastic starch is used to synthesize food packaging materials, disposable utensils, and compostable trash bags. PHAs exhibit immense applications in medical applications including the development of long-term drug release capsules, and tissue scaffolds for neural regeneration (Meereboer et al. 2020). However, certain detailed advantages and disadvantages of bioplastics from various sources are mentioned in Table 1.

## Biodegradation of bioplastics

The biodegradation of bioplastics from various sources depends on their physicochemical properties as well as environmental factors, such as the soil and its essential microbial diversity. Studies have shown that regardless of the source and type of bioplastic, soil enrichment significantly enhances the rate of biodegradation. Additionally, factors such as humidity and temperature play an important role in the biodegradation of bioplastics. (Zoungranan et al. 2020). Further, several components, especially plasticizers, are a necessity while synthesizing bioplastics. Water is the major solvent and plasticizer in biopolymer technology. Apart from water, polyols, mono, di-, and oligosaccharides are the most used plasticizers. Polyols such as glycerol, erythritol, and sorbitol have 3, 4, and 6 carbons and hydroxyl groups, respectively (Lang et al. 2020). It is reasonable to use these organic compounds as plasticizers for biopolymers given their completely biodegradable nature. In addition, the use of plasticizers also assists the quick degradation of bioplastics. In a study, it was revealed that the weight loss of unplasticized PVOH/RWF films was lower than glycerol- and sorbitol-plasticized polyvinyl alcohol/rambutan skin waste flour films (Ooi et al. 2012).

**Fig. 2:**
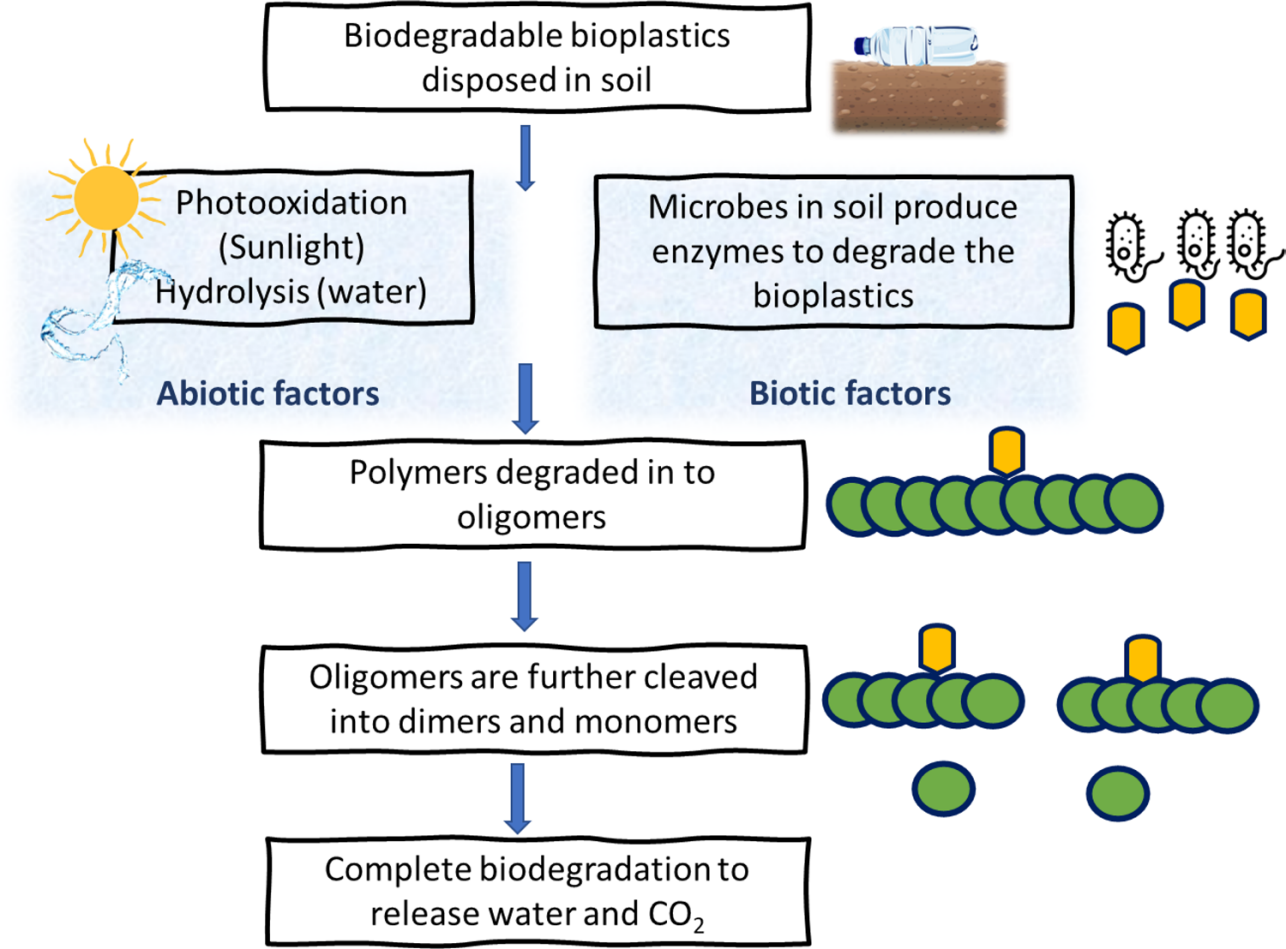
Sequential steps of polymer degradation by microbes in the soil. The figure is adapted from (Kumar Tiwari et al. 2018) with kind permission from Granthaalayah publications

The degradation of the bioplastics with the aid of using soil microorganisms is evident, and numerous microorganisms including *Bacillus sp*. and *Aspergillus sp*., are isolated and recognized as degraders of bioplastics in soil (Adhikari et al. 2016). Table 2 summarizes the common microbes and enzymes responsible for degrading biodegradable bioplastics. Complete biodegradation (≥ 90%) prevents accumulation in soil, which is the initial level of testing biodegradability (Ardisson et al. 2014). The steps involved in the microbial degradation of polymers have been depicted in Fig. 2. However, the rate of biodegradation of bioplastic depends on the soil conditions. The most widespread procedures used to examine the biodegradation of bioplastics in soil are visual analysis, detecting mass loss, spectroscopy, and CO_2_ emission (Zilliges and Damrow 2017; Ruggero et al. 2019; Folino et al. 2020). The volume of CO_2_ produced during biodegradation is used as the index of microbial decomposition. CO_2_ emission can be analyzed using two methods namely the cumulative measurement respirometry (CMR) and gravimetric measurement respirometry (GMR) (Ruggero et al. 2019). Visual analysis techniques include scanning electron microscopy (SEM), macroscopic photographs, and atomic force microscopy (AFM) to examine surface changes in the material. The visual evaluation commonly includes checking for the distribution of particle size in residual bioplastic and the presence of microbial colonization (Marga et al. 2005). Other factors include noticeable degradation phenomena such as loss of consistency, thickness, discoloration, and the presence of physical disintegration like holes or cracks. Further, SEM and AFM are used to thoroughly observe the morphological changes occurring during biodegradation like crack formation, surface roughness, and corrosive degradation (Folino et al. 2020). Another index of studying biodegradation is the percentage of mass loss. It includes measurement of mass loss for pieces of bioplastics extracted after soil burial post the experimental period. The standardized procedure includes sample screening, washing with distilled water, followed by drying and weighing (Salomez et al. 2019).

### Biomass

#### Polysaccharides

Starch is primarily degraded by glycoside hydrolases, enzymes that hydrolyze the glycosidic bonds. The enzyme α-amylase cleaves the long starch polymers, producing smaller fragments that are further hydrolyzed by an array of enzymes including glucoamylase, β-amylase, and α-glucosidase. These enzymes further hydrolyze the α-glycosidic linkages (Encalada 2018). Enzymes that can accomplish starch hydrolysis are present in the soil. Several fungi including *Aspergillus oryzae* and bacteria such as *Klebsiella pneumonia, Bacillus circulans*, and *Bacillus stearothermophilus* synthesize an array of starch hydrolyzing enzymes. Further lytic polysaccharide monooxygenases (LPMOs) produced by a variety of bacteria like *Escherichia coli* and fungi such as *Thermoascus aurantiacus,* and glycoside hydrolases are also efficient in breaking glucose bonds by oxidative cleavage. The biodegradation of thermoplastic starch (TPS) was compared under laboratory conditions (soil and compost) and in the field (soil). Based on microbial activity, it was concluded that fungi have a greater ability to biodegrade TPS than bacteria. It was also observed that both the crystalline and molecular structures of TPS films are factors that influence the enzymatic degradation of TPS by fungal α-amylase. Absolute degradation of TPS films containing higher moisture levels and buried 20 cm deep in soil was observed in 4–6 months (Polman et al. 2021). In another study conducted, two batches of cassava starch films were synthesized, one set crosslinked with citric acid (TPS75-C, heated at 75 ℃ and TPS85-C, heated at 85 ℃) and another without any citric acid crosslinking (TPS7, heated at 75 ℃ and TPS85, heated at 8 ℃) were subjected to biodegradation for 30 days. The degradation process of TPS75 and TPS85 was considerably high in 12 days, while the citric acid cross-linked films (TPS75-C and TPS85-C) required 18 days (Seligra et al. 2016).

**Fig. 3:**
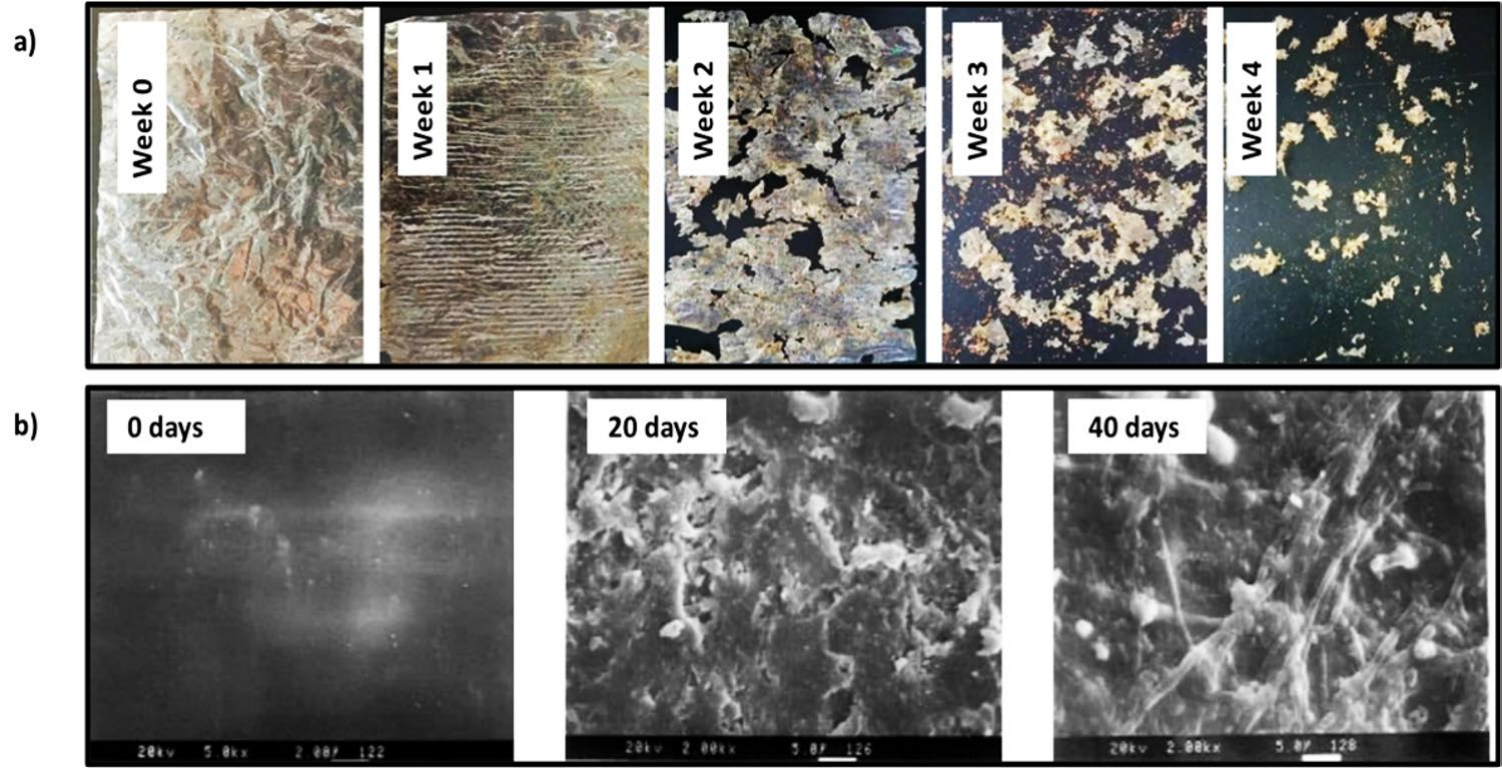
Biodegradability test showing changes in the morphology of cellulose films: **a** Macroscopic images showing degradation of cellulose film over 4 weeks. The image has been reproduced from (Ai et al. 2021) published on Frontiers. **b** SEM images showing biodegradation of regenerated cellulose films from pulps of cotton linter, cotton stalk, and wheat straw over 40 days. The image has been reproduced from (Zhang et al. 1996) with permission from the American Chemical Society

Fungi form the major share of cellulolytic (cellulose-degrading) microorganisms (Tian et al. 2017). Bacteria such as *Pedobacter* and *Mucilaginibacter* revealed intricate enzymatic systems for the degradation of polysaccharides including cellulose and hemicellulose. Cellulases are responsible for extracellular cellulose degradation. These enzymes belong to the glycoside hydrolases and are adept at breaking the β-glycosidic bonds. LPMOs are involved in the initial phase of cellulose biodegradation (Polman et al. 2021). The degradation of cellulose produces cellobiose, a disaccharide of glucose. Depending on the soil condition, cellulose degradation may require 81–495 days. Cellulose acetate (CA) is generated by the acetylation of cellulose. These acetyl groups hinder the microbial and enzymatic attack on the CA-based bioplastics and prolong their time of biodegradation (Hayakawa et al. 2014). However, biodegradation may take place through oxidative cleavage by LPMOs and hydrolysis by cellulases. The collaboration of cellulases, LPMOs, and acetyl esterases is known to degrade CA with a degree of substitution of less than 1.8. The faster degradation of flax fibers was attributed to slight chemical differences between the flax and cotton-based cellulose acetate (CA). Complete breakdown of CA from rice straw was observed after 105 days in the soil. During biodegradation, the degree of substitution of the residual CA remained unaffected, as enzymatic hydrolysis occurs at a higher rate than deacetylation (López-Mondéjar et al. 2016). In a study, regenerated cellulose films were prepared from cellulose solution from pulps of cotton stalk, cotton linter, and wheat straw. They were buried in the soil to assess biodegradability (Fig. 3a). Results indicated 10% weight loss for film in 16 days (Zhang et al. 1996). In another study, cellulose film was observed to be decomposed in soils within only 4 weeks (Fig. 3b), suggesting an exceptional biodegradability as compared to the polyethylene plastic film which demonstrated no indications of degradation (Ai et al. 2021).

**Fig. 4:**
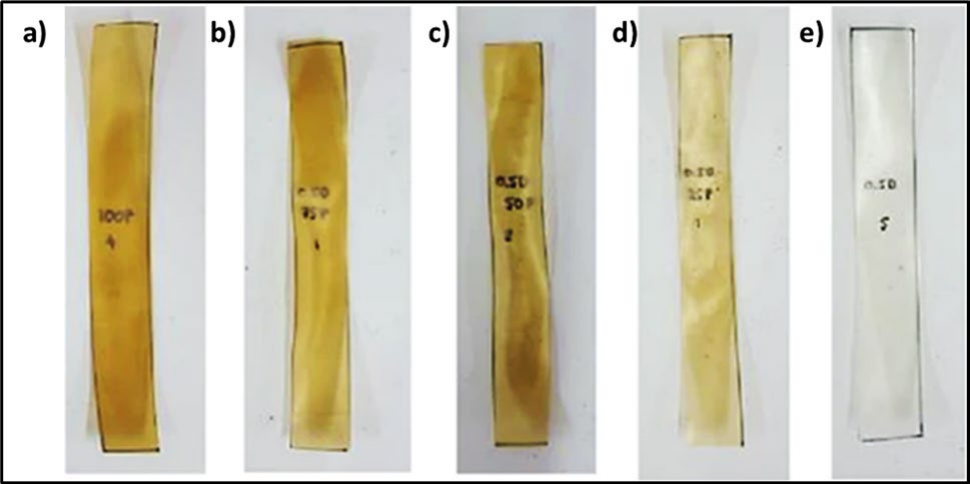
Digital macroscopic images for high-methoxy pectin (HMP) film with varying concentrations of DS films after burial in soil for 15 days: **a** 100HMP/0DS, **b** 75HMP/25DS, **c** 50HMP/50DS, **d** 25HMP/75DS, and **e** 0HMP/100DS. The image has been adapted from (Bátori et al. 2017) with permission from Hindawi

In a study, it was found that a thermoplastic starch strengthened with 50% wt. lignocellulosic fibers and flax fibers degraded considerably slower than those of native palm, banana, and bagasse (sugar cane residue) (Jumaidin et al. 2021). Pectin is degraded by a group of enzymes called pectinases (Table 2). These enzymes work by depolymerization and de-esterification of the pectin. Pectinolytic bacteria such as *Erwinia* spp. degrade pectin by producing such enzymes (Abbott and Boraston 2008). The anaerobic degradation study of pectin-cellulose biofilms from orange peel wastes exhibited 90% degradation in about 15 days. A highly biodegradable high-methoxy pectin (HMP) film with dialdehyde starch (DS) (0%, 25%, 50%, 75%, 100%) was developed. Increasing the content of DS caused a decrease in the biodegradation percentage (Fig. 4) (Bátori et al. 2017).

**Fig. 5:**
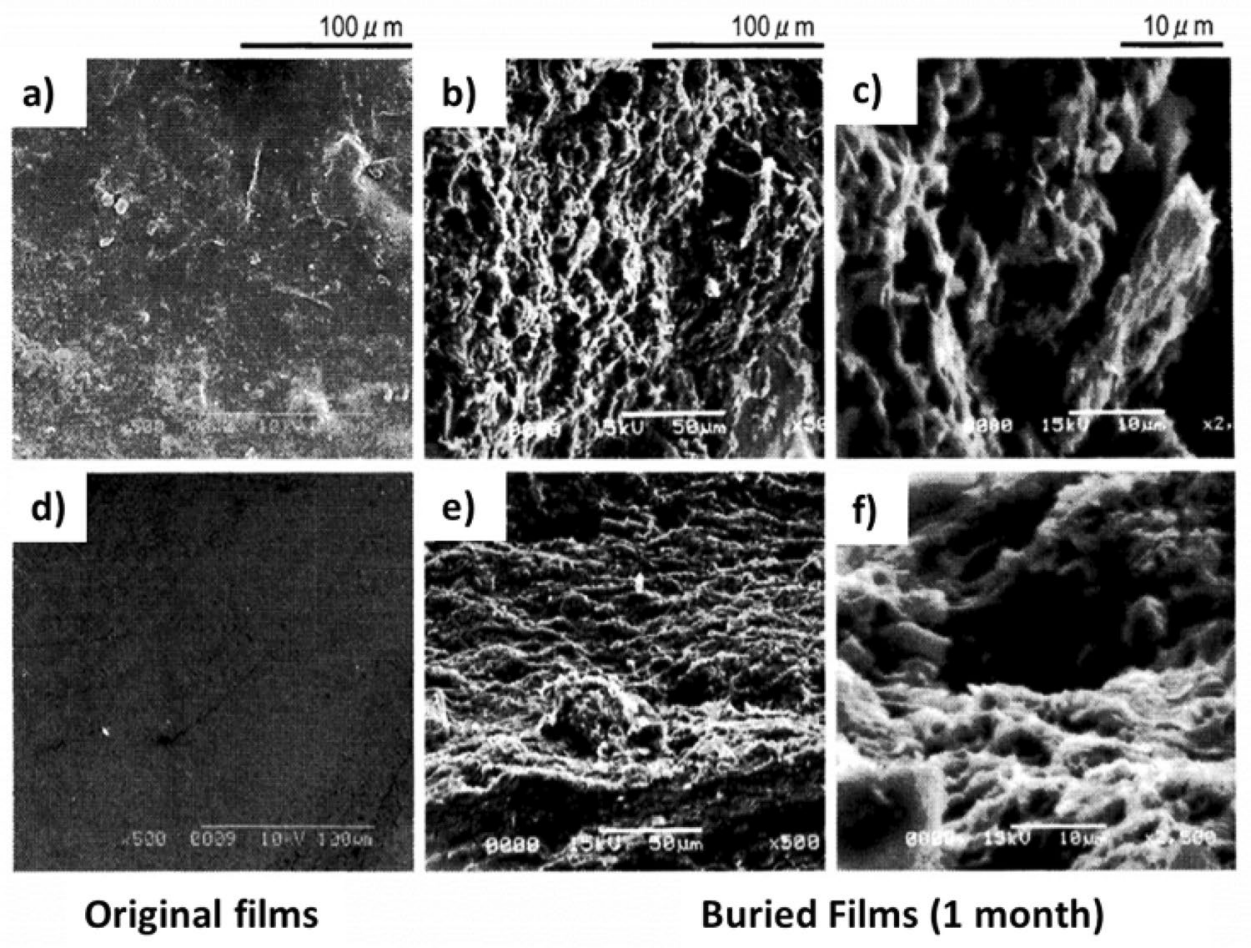
SEM images of **a**, **b**, **c** chitin films and **d**, **e**, **f** chitosan films buried for 1 month in red clay. **a** and **d** SEM images of chitin and chitosan films, respectively, before soil burial. **b** and **c** SEM images of chitin films; **e** and **f** SEM images of chitosan films after a month of burial in red clay. The images have been reproduced from (Nakashima et al. 2005) with kind permission from J-Stage

Chitin and chitin-based bioplastics are degraded by the action of bacteria and fungi in soil by a process called, chitinolysis via chitinases. Chitin undergoes deacetylation to produce chitosan, which is hydrolyzed by chitosanases (Gooday 1994). Bacterial species of*Aeromonas, Vibrio, Cytophaga, Photobacterium, Streptomyces, Bacillus, Chromobacterium,* and *Clostridium* are well-known chitinolytic bacteria. Chitinolytic fungal species include Mucorales like Deuteromycetes and *Mortierella* spp, and Ascomycetes like *Aspergillus*, *Thielavia, Trichoderma, Humicola Penicillium,* and *Verticillium* (Moussian 2019). The biodegradation of chitin and chitosan films revealed that after 1, 1.5, and 2 months, chitin films buried in red clay achieved 83.8%, 99%, and 100% weight loss, respectively. In the case of chitosan films, the weight loss was 79.2%, 98.9%, and 100% for the same periods and conditions. (Nakashima et al. 2005). Figure 5 exhibits the SEM images of chitin and chitosan films before and after biodegradation. In another study, the biodegradation of two composite films polyethylene-chitin (PE-chitin) and polyethylene-chitosan (PE-chitosan) films, containing 10% chitin or chitosan, by pure microbial cultures in a soil environment was studied and compared to commercial starch films. In soil, 73.4% of the PE-chitosan and 84.7% of PE-chitin degraded, while only 46.5% of the commercial starch film degraded after six months (Lee 1995).

**Fig. 6:**
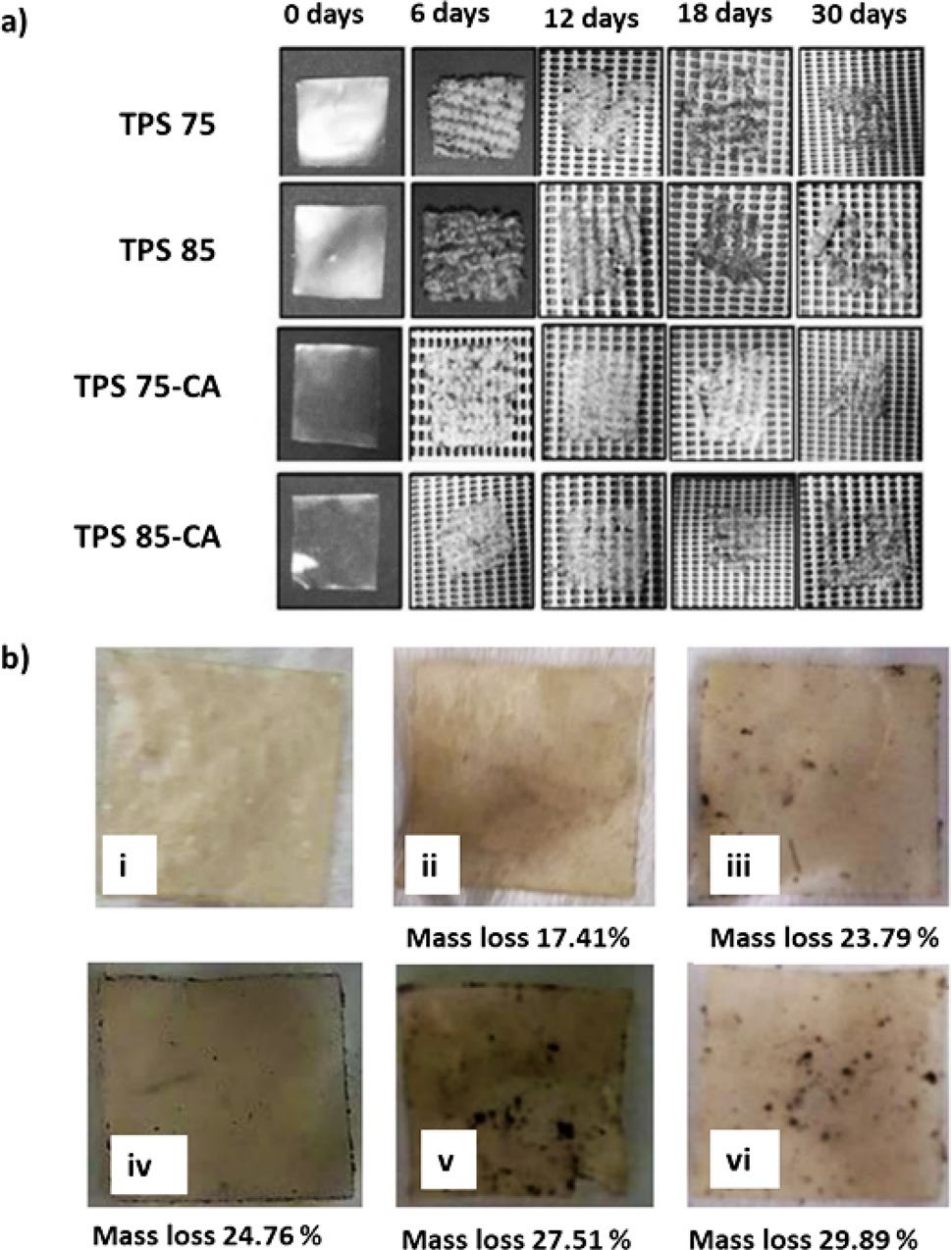
**a** Macroscopic appearance of thermoplastic starch (TPS) films biodegradation in soil for 30 days. The figure has been reproduced from (Seligra et al. 2016) with kind permission from Elsevier. **b** Macroscopic images of soil burial test of starch-based bioplastic over 10 days indicating respective mass loss%: (i) Day 0, (ii) Increasing size of bioplastic on day 2, (iii) More soil water and the soil itself have entered the bioplastic pores on day 4, (iv) Wider cracking area on bioplastic sample on day 6, (v) Some parts of bioplastic were destroyed on day 8, (vi) Wider parts of the bioplastic were destroyed on day 10. The figure has been reproduced from (Nissa et al. 2019) with kind permission from IOP science

It was observed that starch-based bioplastic biodegrades much faster compared to bioplastics developed from other polysaccharides such as cellulose (Table 3) based on the prevailing conditions including the microbial flora. This may be attributed to the fact that cellulose as a polysaccharide is much more stable compared to starch due to its molecular orientation where opposite molecules are placed at a rotation of 180° from each other forming a rigid elongated structure (Conley et al. 2016). However, the rate of bioplastic biodegradation is faster in a composting plant compared to natural conditions due to the continuous supply of air as well as humidity and temperature control. The biodegradation of starch-based bioplastics in the soil is depicted in Fig. 6. However, several factors such as the presence of additives affect the time taken for the degradation of bioplastics.

#### Proteins of plant and animal origin

**Fig. 7:**
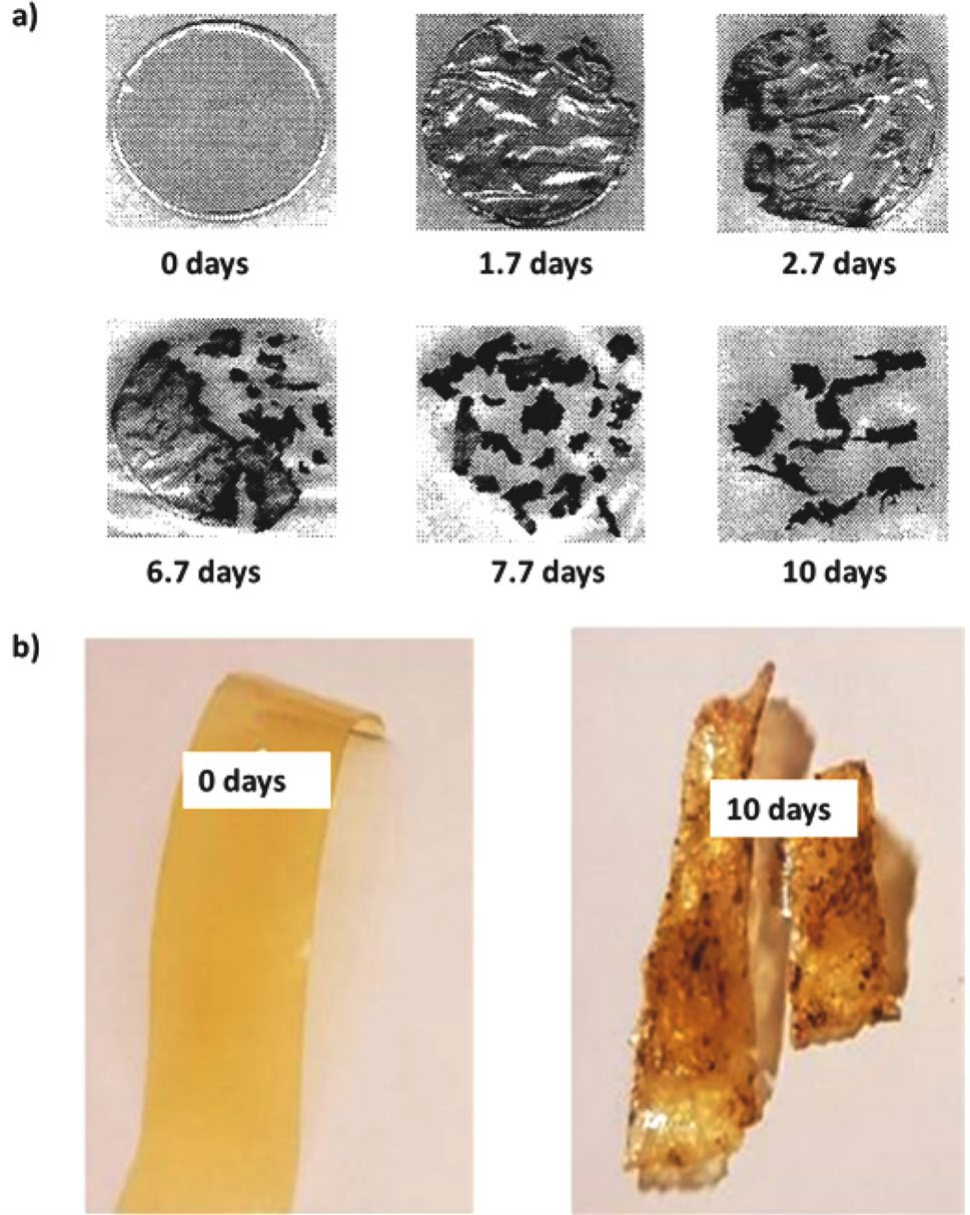
**a** Digital images of whey protein isolate films before and after soil composting burial test over 10 days. The images have been reproduced from (Li and Chen 2000) with permission from Springer Nature. **b** Macroscopic digital images showing biodegradation of hydrophobic zein-based functional films over 10 days. The images have been reproduced from (Luís et al. 2019) with kind permission from MDPI

According to a study conducted by Bagares et al., the degradation of rapeseed protein-based bioplastic was 57% and 74% in the soil and liquid medium, respectively. The bioplastic produced from casein-based bioplastic from spoiled processed cow’s (Bos taurus) milk took seven days to be degraded with the help of catalysts such as scavenger insects in the presence of sunlight and rain (Bagares et al. 2020). Further, the transparent whey protein isolate films began to degrade within only 2 days and completely degraded over 7 days with more than 80% of weight loss. Figure 7a shows a scanned image of whey protein isolate films after 10 days of soil burial. Similarly, there are reports of soy protein and whey protein isolate composite films being degraded in about 7 days with 36% of weight loss (Li and Chen 2000). In another study, hydrophobic zein-based bioplastic films containing licorice essential oil were developed (Luís et al. 2019). The biodegradability of the films was analyzed by soil burial degradation test for 10 days (Fig. 7b). The films lost about 50–60% of their weight, which is a strong indication of the biodegradation process carried out by the microorganisms in the soil. The visual analysis confirmed that, after 10 days in the soil, the films appeared thinner and more fragmented compared to the initial samples (Luís et al. 2019). Wheat gluten-based bioplastics were subjected to biodegradation in farmland soil. All gluten-based materials were entirely degraded within 50 days in farmland soil (Domenek et al. 2004).

The study of biodegradation of bioplastics from biomass such as proteins, polysaccharides (starch, cellulose, lignin chitin, and pectin), microorganisms, and bioderived as well as synthetic monomers receive less attention when compared to physicochemical properties of these materials. Further, the comparison of biodegradation of modified composite polymers with one or more than one component from biomass has been studied in this review (Table 3). It is generally observed that animal-based protein bioplastics are easier to break down compared to plant-based protein bioplastics since plant proteins lack branched chain amino acids (BCAA).

#### Polyesters of bacterial origin

**Fig. 8:**
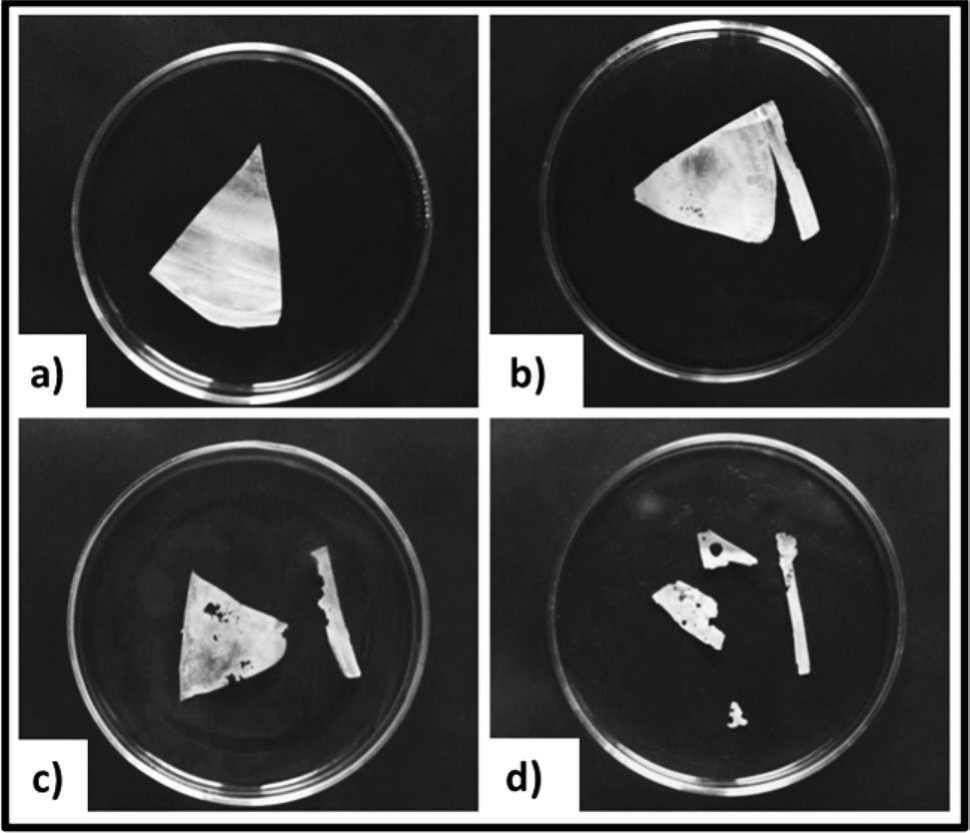
**a** Undegraded PHB film and PHB films with different degrees of degradation after 2 months of incubation in experimental flasks **b** 0% O_2_ and 0 g/L ${\mathrm{NO}}_{3}^{-}$ **c** 10% O_2_ and 0 g/L${\mathrm{NO}}_{3}^{-}$, and **d** 10% O_2_ and 5 g/L ${\mathrm{NO}}_{3}^{-}$. The image has been reproduced from (Bonartseva et al. 2002) with permission from Springer Nature

Microbes that produce extracellular PHA-degrading enzymes are widespread in the soil and marine environment. PHAs are biodegraded by the action of bacteria present in the soil. These bacteria belong to the following genera: *Arthrobacter, Corynebacterium, Actinomyces, Acinetobacter, Alcaligenes, Aspergillus, Bacillus, Burkholderia, Clostridium, Comamonas, Cupriavidus, Mycobacterium, Pimelobacter, Enterobacter, Gracilibacillus, Planococcus, Nocardia, Pseudoalteromonas, Staphylococcus, Micrococcus, Klebsiella, Streptomyces, Pseudomonas, Stenotrophomonas, and Variovorax* (Trivedi et al. 2016)*.* PHA-degrading fungi are reported to be more efficient and belong to the divisions Ascomycota, *Basidiomycetes, Deuteromycetes*, and *Zygomycotina*. Biodegradation of PHA in soil is facilitated by lipases and hydrolases (Table 2). The process involves breaking down the polymer into oligomers by hydrolytic depolymerase in the presence of water, converting PHAs into trimer and dimer units, which are then treated by lipases and hydrolases. (Meereboer et al. 2020). PHB depolymerases can be secreted by several microorganisms. Some PHB depolymerases were isolated and purified from microorganism *species Alcaligenes, Comamonas,* and *Pseudomonas* (Suzuki et al. 2021)*.* The biodegradation of starch/PHB-V blends has been studied on several occasions. Accinelli et al. developed a composite bioplastic synthesized from a combination of corn starch and poly(3-hydroxybutyrate)-co-poly (3-hydroxy valerate) (PHB-V) in various proportions. The biodegradability of the developed composite bioplastics in soil compost was assessed for 10 months. PHB-V blends with 50% of starch enhanced the biodegradability causing it to degrade within 50% of the time required for PHB-V bioplastic without any starch (Accinelli et al. 2012). Further, three types of TPS synthesized from potato, corn, and water-soluble potato starch, blended with PHB were evaluated in a soil burial test. The mass loss increased with increasing time and increasing glycerol content added during the synthesis of bioplastic but decreased with increasing content of PHB (dos Santos et al. 2018b, a). The biodegradation of PHB films in soil was studied including and excluding nitrate and at varying concentrations of oxygen. It was observed that the PHB film completely degraded in two months under aerobic conditions in the presence of nitrate in the soil. (Fig. 8) (Bonartseva et al. 2002).

### Monomers

#### Bio-derived monomers

**Fig. 9:**
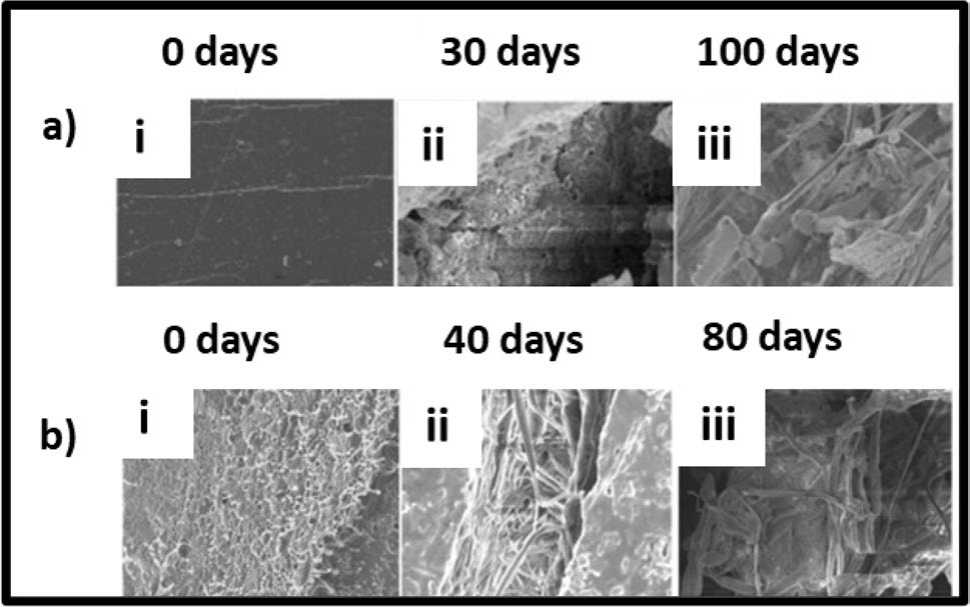
FESEM images representing **a** PLA and **b** PLA-Chitosan composite films before and after biodegradation under composting conditions over 100 and 80 days, respectively The images have been reproduced from (Kalita et al. 2020, 2021) with permission from Elsevier

PLA has garnered significant interest in biomedical and packaging applications due to its biodegradable and hydrophobic characteristics. The cleavage of ester bonds is the primary reason behind PLA degradation. Polymer degradation can be further caused by an array of factors, including oxidation, photodegradation, thermolysis, hydrolysis, and biodegradation. The biodegradability of PLA in soil is dependent on microorganisms (bacteria, fungus) and biochemical processes of degradation (Weng et al. 2013). During biodegradation, PLA is first hydrolyzed and then decomposed by microorganisms into carbon dioxide and water. Primary PLA degrading enzymes include lipase, esterase, and alcalase (protease). PLA can be degraded in soil, due to the presence of many bacterial species such as *Saccharothrix, Kibdelosporangium, Pseudonocardia, Lentzea,* and *Amycolatopsi*. In addition, studies reported that under composting conditions in forest soil consisting of a mixture of *Actinomadura keratinilytica* and *Thermopolyspora sp*, PLA film samples in soil were degraded completely in three weeks (Zaaba and Jaafar 2020). Researchers found that the addition of starch augmented the biodegradation of the PLA-based bioplastics. SEM study revealed crack formation after biodegradation caused due to the action of microbes on the surface of the polymers (Qi et al. 2017; Kalita et al. 2020). In another study, the field emission scanning electron microscope (FESEM) images of PLA-chitosan composite film after composting showed massive morphological changes on the 40th and 80th days of composting process in soil. Microbial assimilation during the biodegradation developed holes and cracks on the polymer surface, cross-section, and core (Kalita et al. 2021) (Fig. 9). PLA bioplastics degrade extremely slowly, due to increased hydrophobicity of the methyl group on the lactide monomer and prevent the degrading enzymes from penetrating the polymer and breaking the existing ester bond (Farah et al. 2016).

#### Synthetic monomers

**Fig. 10:**
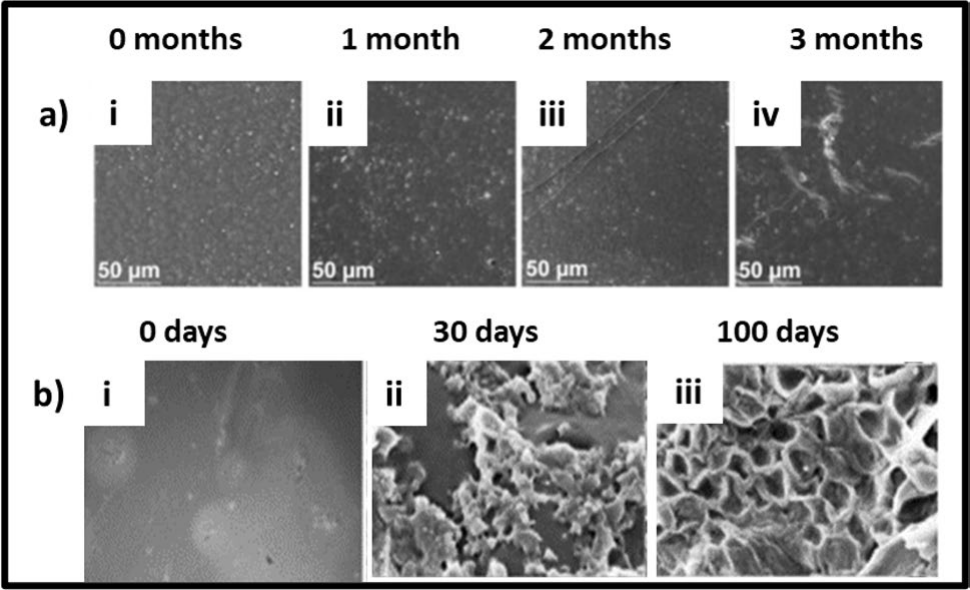
**a** SEM images of PBAT film surface before and after the soil burial test: (i) 0 months (ii) after 1 month (iii) after 2 months (iv) after 3 months. This image has been reproduced from (Wang et al. 2015) with kind permission from Elsevier. **b** FESEM images representing the PCL film samples before and after biodegradation over 100 days under composting conditions. The image has been reproduced from (Kalita et al. 2020) with permission from Elsevier

Aliphatic-co-aromatic polyesters are hard to be degraded by enzymatic hydrolysis, but many enzymes that degrade PBAT have been found among carboxylic ester hydrolases, including carboxylesterase, arylesterase, triacylglycerol lipase, and cutinases (Table 2) (Kawai et al. 2019). The rate of biodegradation of PBAT can fluctuate from 5 to 95% subject to conditions of biodegradation used (composting, soil, enzymatic tests, etc.) (Feuilloley et al. 2005). PBAT films were reported to biodegrade at unique rates in food, manure, and yard compost with different microbial activities. The maximum biodegradation rate was found in manure compost, resulting in the highest CO_2_ emission. SEM studies reveal that undegraded PBAT film shows a smooth surface (Weng et al. 2013). With increasing time of soil burial and the effects of microorganism activities, microbe-created cavities were observed on the surfaces (Wang et al. 2015). PCL surface morphology was rough with certain fibrillar structures (Kalita et al. 2020). PCL is one of the few synthetic biodegradable polymers available and is used by microorganisms as an energy and carbon source (Chu and Wang 2013). The enzymes, lipase, and cutinase belong to the esterases under the hydrolytic enzymes. Recently, a combination of these two enzymes proved to be appropriate for the biodegradation of PCL (Liu et al. 2019a). PCL is biodegraded by cutinolytic enzymes secreting bacteria like *Pseudozyma japonica*-Y7-09 (Abdel-Motaal et al. 2014). Further, a thermophilic *Streptomyces thermoviolaceus subsp. thermoviolaceus* isolate 76 T-2 isolated from soil in Taiwan is reported to cause biodegradation of PCL (Chua et al. 2013). An early study reported that soil burial and compost cause chain scission of the PCL backbone. This results in loss of mechanical properties and significant weight loss of PCL bioplastics in a short time (Ardisson et al. 2014). Lipases from *R. delemar, R. arrizus, Candida cylindracea*, and Achromobacter sp. and esterase from hog liver showed degrading activities on PEA and PCL. Studies showed that PCL completely degraded in 12 days using *Penicillium* sp. strain 26–1 (ATCC 36507) isolated from soil. Figure 10 depicts FESEM images of biodegradation of PCL film.

The most common approach in the literature is to examine the total mass loss. Table 3 compares the percentage mass loss of bioplastics after soil burial under different conditions. For a bioplastic to be considered truly biodegradable, it must degrade into carbon dioxide, water, biomass, and/or mineral salts when exposed to air, moisture, soil, and microbes. Bio-based materials can also be used as plasticizers and reinforcements for bioplastic polymers, affecting their rate of biodegradation while also improving their functional properties.

## Conclusion

Plastics are used in our daily lives as packaging materials, scientific gadgets, and in a variety of other applications. However, traditional plastics are non-biodegradable, and their rapid buildup in the soil ecosystem poses a hazard to the environment. Although plastics have significantly improved our quality of life, it is crucial to shift toward sustainable alternatives, such as bio-based biodegradable plastics. Biodegradable plastics are gaining popularity, and for the sake of the environment, plastics could be replaced by biodegradable bioplastics derived from various sources. The biodegradation of bioplastics in the soil can be affected by numerous elements which include microbial diversity, temperature, humidity, and pH of the soil. The most common techniques in the literature to study the biodegradation of plastics are mass loss and visible evaluation via macroscopic digital and SEM images. Biomass, particularly polysaccharides like starch and cellulose, is widely preferred for the synthesis of bioplastics. However, the increasing demand for such bioplastics may create heavy competition for food sources. Starch and cellulose are commonly used polysaccharides. Also, starch is more hygroscopic than cellulose, making starch-based bioplastics more susceptible to degradation. Additionally, discarded bioplastics in landfills release methane, a potent greenhouse gas, which could negatively impact the ecosystem. Bioplastics of microbial origin have a significant constraint in the form of bulk manufacturing and high recovery costs. Bioplastics may be implemented in the manufacturing of scientific devices and various other important applications including flexible packaging. Biodegradability is a characteristic regularly sought in terms of food packaging. Due to the extensive variety of bioplastics from diverse sources, the life cycle or the time required for complete biodegradation of every bioplastic differs, relying on situations of biodegradation and available waste control systems in different places. In the recent times of the COVID-19 pandemic, the usage of plastics has been aggravated by the immoderate use and intake of single-use plastics (consisting of personal protective equipment like masks and gloves). With these current traits of expanded utilization of conventional non-biodegradable plastic in packaging, food, medical, and health industry, it is critical to update the single-use plastics with eco-friendlier options.

## Data Availability

Data availability not applicable.
